# Necrotizing Fasciitis of the Nose Complicated with Cavernous Sinus Thrombosis

**DOI:** 10.1155/2014/914042

**Published:** 2014-04-30

**Authors:** D. Swaminath, R. Narayanan, M. A. Orellana-Barrios, B. Temple

**Affiliations:** Department of Internal Medicine, Texas Tech University Health Sciences Center, Lubbock, TX 79430, USA

## Abstract

Necrotizing fasciitis is a rapidly progressive life threatening bacterial infection of the skin, the subcutaneous tissue, and the fascia. We present a case of necrotizing fasciitis involving the nose complicated by cavernous sinus thrombosis. Few cases of septic cavernous sinus thrombosis have been reported to be caused by cellulitis of the face but necrotizing fasciitis of the nose is rare. It is very important to recognize the early signs of cavernous thrombosis. Treatment for septic cavernous sinus thrombosis is controversial but early use of empirical antibiotics is imperative.

## 1. Introduction

Necrotizing fasciitis is a rapidly progressive life threatening bacterial infection of the skin, the subcutaneous tissue, and the fascia [1]. The trunk and extremities are the most common sites of infection but rarely face and neck regions can be involved; due to its rarity, involvement of the face requires a high level of clinical suspicion. Early diagnosis and aggressive surgical intervention coupled with systemic antibiotic treatment are essential for better outcome and decreased mortality [1]. When the face is included, these interventions might prevent complications such as meningitis and cavernous sinus thrombosis. In this case report we present a case of necrotizing fasciitis involving the nose complicated by cavernous sinus thrombosis.

## 2. Case Presentation

The patient is a 42-year-old male with past medical history of systemic hypertension and depression, complaining of facial swelling and fever for two days. The facial swelling started after he pulled “a hair” from his left nostril three days before. The swelling progressively worsened, extending to both nostrils and around his eyes. He also complained of constant severe pain in and around his nose, rated 9/10, relieved with analgesics. At an outside facility he had a lumbar puncture and head CT scan performed, started on vancomycin, and then transferred to Texas Tech University Hospital.

**Figure 1:**
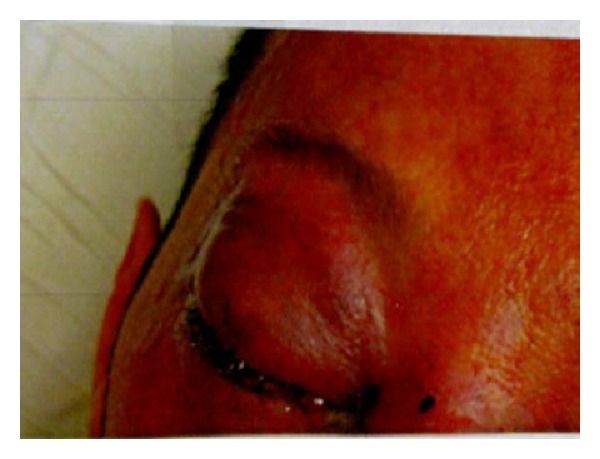
Right eye preseptal cellulitis showing severe chemosis with yellowish crust in the eyelids.

On arrival he had a temperature of 103°F (39.4°C) and was awake, alert, and oriented with mild distress secondary to pain. He was complaining of eye swelling, nasal pain, and congestion. Erythema and swelling on his forehead, nose, eyelids, periorbital regions, and perinasal regions were noted. Eye examination revealed intact extraocular muscle and severe chemosis but intact vision with yellowish crust over his eyelids. There was reduced adduction of the right eye in the direction of action of the inferior oblique, but otherwise normal III cranial nerve function. Also, examination of cranial nerves IV, VI, V1 and V2 was normal as was the rest of the eye exam. Severe tenderness was noted on palpation of the nose with erythema and bilateral swelling of the mucus membranes (Figure 1). All other exams were normal.

His cerebral spinal fluid (CSF) results from the previous facility showed a white blood cell count (WBC) of 1,197/mm^3^, RBC 27/mm^3^, protein 100 mg/dL, and glucose of 54 mg/dL. Initial labs revealed a peripheral blood WBC of 24,000/uL with a predominance of neutrophils (85%). The CT scan of the maxillofacial area showed a fluid collection with multiple small air loculi in the anterior nasal cavity as well as evidence of possible osteomyelitis of nasal septum and a component of acute sinusitis in right frontal sinus. Ear, nose, and throat (ENT) surgery, infectious diseases (ID), neurosurgery, and neurology were consulted and the patient was started on empirical antibiotic (vancomycin and ceftriaxone).

Incision and drainage of the septal abscess with washout and debridement of the nasal septum using nasal endoscopy were performed; tissue necrosis was noted during the procedure. ID replaced the ceftriaxone with clindamycin (for possible toxin production) and meropenem for broader gram-negative and anaerobe coverage.

**Figure 2:**
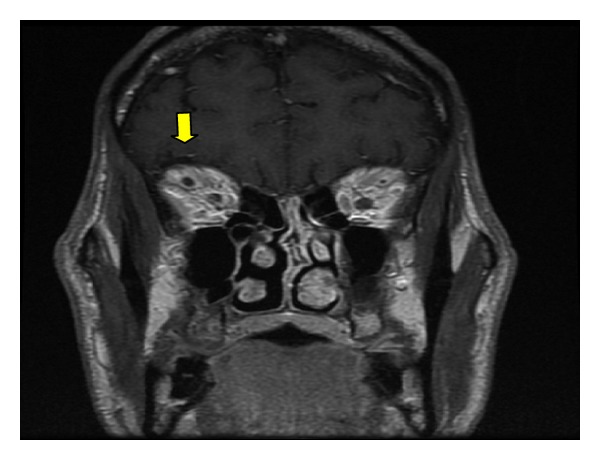
Head MRI with enlarged right superior ophthalmic vein.

**Figure 3:**
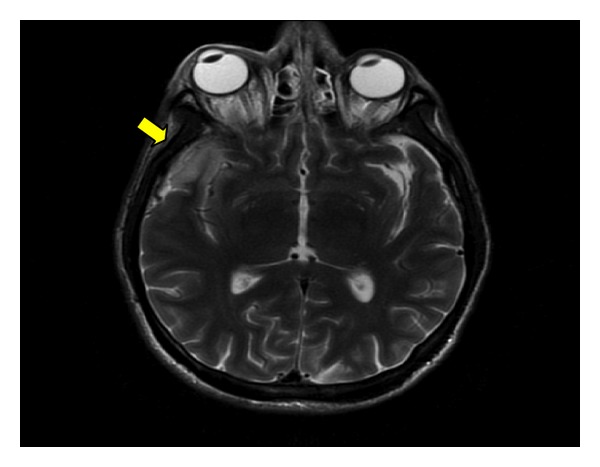
Head MRI T2w image showing hyperintensity in the right anterior temporal lobe.

Magnetic resonance imaging (MRI) and magnetic resonance venography (MRV) (Figures 2-3) studies demonstrated concerns for cavernous sinus thrombosis. A MRI with contrast failed to reveal filling defect of the cavernous sinus but noted mild proptosis of the right orbit and dilatation of the right superior ophthalmic vein, suggestive secondary signs of right cavernous sinus thrombosis.

Prior to discharge he had improving mild right-sided ptosis but was otherwise asymptomatic and afebrile. As per neurosurgery and neurology recommendations, no anticoagulation was needed for the cavernous sinus thrombosis. His final incision and drainage cultures were positive for methicillin resistant* Staphylococcus aureus* (MRSA) with a vancomycin minimum inhibitory concentration (MIC) equal to 2.0. His antibiotics were changed to IV daptomycin for 6 weeks as per infectious diseases recommendations. He continued to receive irrigation of the nares with gentamicin and vancomycin as per ENT surgery's recommendations. He was discharged on IV daptomycin only and followed up by surgery in the outpatient clinic. There were no neurological deficits or recurrent infection during the 2-month follow-up period.

## 3. Discussion

Skin and soft tissue infections (SSTIs) account for more than 14 million outpatient visits in USA each year. Not only the outpatient visit has increased but also hospital admissions for SSTIs increased by 29% from 2000 to 2004 [1]. Necrotizing fasciitis is a well-known complication of untreated and poorly treated cellulitis of skin and soft tissue infection. Necrotizing infections warrant prompt aggressive surgical debridement.

Strongly suggestive clinical signs include bullae, crepitus, gas on radiography, hypotension with systolic blood pressure less than 90 mm Hg, or skin necrosis [1]. As in our case the patient was hypotensive on initial presentation with necrosis noted during endoscopy, a characteristic finding of the necrotizing fasciitis. These are late findings, seen in fewer than 50% of cases. Most cases of necrotizing fasciitis have a false admission diagnosis of cellulitis.

The cavernous sinus receives drainage from cortical and deep cerebral veins and also from the sinus systems of the meninges, scalp, and nasal sinuses, facilitating the spread of infection and/or thrombosis between these vessels. Conversely, a French study of 62 adults with isolated lateral sinus thrombosis found that only 3 cases were related to parameningeal infections [2]. Cavernous sinus thrombosis is an uncommon condition with a varied and often dramatic clinical presentation [3]. These clinical presentations are due to sinus obstruction and impairment of the cranial nerves that are run in close proximity to cavernous sinus [3].

Headache is the most common symptom, usually preceding fevers, periorbital edema, and cranial nerve signs [4]. Physical examination reveals periorbital edema and chemosis, lateral gaze palsy (isolated cranial nerve VI) [4]. CN VI palsy presents early due to its anatomical location (CN VI lies freely within the cavernous sinus), in contrast to CN III and IV, which lie within the lateral walls of the sinus. Ptosis, mydriasis, and eye muscle weakness from cranial nerve III dysfunction may ensue as the disease progresses [4]. Meningeal signs or systemic signs indicative of sepsis are late findings of the disease. In our patient we noted the periorbital edema, limitation of extraocular movement, signs of sepsis, and diagnostic imaging indicating high suspicion of CVS thrombosis.

The American Heart Association (AHA)/American Stroke Association (ASA) 2011 Scientific Statement recommends imaging of the cerebral sinus system in patients with suspected cerebral sinus thrombosis [5]. When clinically suspected the primary modality of investigation includes CT and MRI of the brain. Conventional venography which has higher sensitivity is not used because of increased concerns of dissemination of infection and thrombus extension in patients with septic thrombophlebitis. Cerebral angiography is reserved for the definitive assessment of intracavernous aneurysms after detection and monitoring by CT or MRI. Gallium scintigraphy has occasionally been used as a confirmatory tool in septic CST, demonstrating increased uptake in the cavernous sinus and affected orbit [5]. Our patient did have orbital venous congestion MRI/MRV findings concerning cavernous sinus thrombosis. However, enlargement of the superior ophthalmic vein with proptosis mostly of the right eye was noted. Also, there was evidence for significant mucoperiosteal thickening and sinus disease involving the sphenoid sinuses, although definite filling defects in the cavernous sinus were not seen in our patient. Diagnosis of necrotizing fasciitis requires high clinical suspicion with supportive laboratory/imaging studies. MRI and CT scanning are the recommended diagnostic imaging modalities.

Immediate empiric antibiotic coverage for facial fasciitis must include gram-positive, gram-negative, and anaerobic bacteria coverage and can be narrowed as cultures and sensitivities become available. Surgical intervention of the primary source of infection, including the nasal septum, nasal sinus, and face, is the primary treatment modality. Necrotizing fasciitis needs surgical debridement and concomitant broad-spectrum antibiotic therapy. Empirical antimicrobial therapy usually depends on the antecedent clinical condition and must include coverage for MRSA. An empirical combination, such as parenteral metronidazole, vancomycin, and ceftriaxone, will achieve reasonable CSF and brain penetration and is likely to be active against* S. aureus* (including CA-MRSA strains) as well as the usual sinus pathogens [6]. After the initial empirical antibiotics it is important to deescalate antibiotic [6]. Antimicrobial selection can be based on positive cultures which are subsequently obtained from blood or CSF although care should be taken, as sinus infections may be polymicrobial. Duration of parenteral antimicrobial therapy should be at least 4 weeks.

The role of anticoagulation as an adjuvant to antibiotic therapy remains controversial, as risk of intracranial bleeding and benefit in preventing further thrombotic proliferation need to be assessed [7, 8]. The primary goal of therapy with anticoagulation is to prevent thrombus propagation, recanalize occluded sinuses and cerebral veins, and prevent complications of deep vein thrombosis and pulmonary embolism. Immediate anticoagulation is administered with either intrasinus unfractionated heparin or with subcutaneously administered low-molecular weight heparin as a bridge to oral anticoagulation with a vitamin K antagonist. Even though feared complication of anticoagulation is intracranial bleeding, in a retrospective study of 102 cases treated with heparin, there was no significant increase in cerebral hemorrhage even in those with preexisting bleeds at presentation [9]. Anticoagulation has been controversial for treatment of cerebral sinus thrombosis because of the tendency for sinus infarcts to become hemorrhagic even before anticoagulants have been administered [9].

Implication of outcome is as follows. Although a thorough discussion of necrotizing fasciitis or cavernous vein thrombosis is beyond the scope of this report, we present an interesting and life threatening condition [10]. As previously stated, high clinical suspicion for septic cavernous venous thrombosis, immediate surgical drainage, and appropriate empirical antibiotic therapy with broad-spectrum coverage are crucial in improving the outcome. As anticoagulation for sinus thrombosis is still controversial and our patient had a good outcome after antibiotics, he was not put on warfarin therapy.

Cavernous sinus thrombosis as a complication of necrotizing fasciitis of the nose is a rapidly progressive and dangerous condition that requires immediate initiation of intensive treatment, including broad-spectrum antibiotics, surgical drainage of the source of infection, anticoagulants, and possibly steroids [10]. Initial clinical suspicion and early MRI use are necessary for the accurate diagnosis of extension of intracranial complications. This case demonstrates a rare presentation of nasal cellulitis with cavernous sinus thrombosis. The guarded prognosis for septic CST in the antibiotic era may be primarily due to delays in recognition of this condition and delays in initiating treatment, as well as other complications developing concomitantly with septic CST.
